# Integrative genetic and hormonal coordination of endocarp/stone development in drupe fruit

**DOI:** 10.1093/hr/uhag178

**Published:** 2026-05-26

**Authors:** Bilal Ahmad, Liu Huawei, Vivek Yadav, Ren Shuxian, Yuan Yu, Daoyuan Zhang, Zongrang Liu

**Affiliations:** State Key Laboratory of Desert and Oasis Ecology, Key Laboratory of Ecological Safety and Sustainable Development in Arid Lands, Xinjiang Institute of Ecology and Geography, Chinese Academy of Sciences, Urumqi, China; Xinjiang Key Lab of Conservation and Utilization of Plant Gene Resources, Xinjiang Institute of Ecology and Geography, Chinese Academy of Sciences, Urumqi, China; State Key Laboratory of Desert and Oasis Ecology, Key Laboratory of Ecological Safety and Sustainable Development in Arid Lands, Xinjiang Institute of Ecology and Geography, Chinese Academy of Sciences, Urumqi, China; Xinjiang Key Lab of Conservation and Utilization of Plant Gene Resources, Xinjiang Institute of Ecology and Geography, Chinese Academy of Sciences, Urumqi, China; Institute of Fruits and Vegetables, Xinjiang Academy of Agricultural Sciences, Urumqi, China; State Key Laboratory of Desert and Oasis Ecology, Key Laboratory of Ecological Safety and Sustainable Development in Arid Lands, Xinjiang Institute of Ecology and Geography, Chinese Academy of Sciences, Urumqi, China; Xinjiang Key Lab of Conservation and Utilization of Plant Gene Resources, Xinjiang Institute of Ecology and Geography, Chinese Academy of Sciences, Urumqi, China; State Key Laboratory of Desert and Oasis Ecology, Key Laboratory of Ecological Safety and Sustainable Development in Arid Lands, Xinjiang Institute of Ecology and Geography, Chinese Academy of Sciences, Urumqi, China; Xinjiang Key Lab of Conservation and Utilization of Plant Gene Resources, Xinjiang Institute of Ecology and Geography, Chinese Academy of Sciences, Urumqi, China; State Key Laboratory of Desert and Oasis Ecology, Key Laboratory of Ecological Safety and Sustainable Development in Arid Lands, Xinjiang Institute of Ecology and Geography, Chinese Academy of Sciences, Urumqi, China; Xinjiang Key Lab of Conservation and Utilization of Plant Gene Resources, Xinjiang Institute of Ecology and Geography, Chinese Academy of Sciences, Urumqi, China; State Key Laboratory of Desert and Oasis Ecology, Key Laboratory of Ecological Safety and Sustainable Development in Arid Lands, Xinjiang Institute of Ecology and Geography, Chinese Academy of Sciences, Urumqi, China; Xinjiang Key Lab of Conservation and Utilization of Plant Gene Resources, Xinjiang Institute of Ecology and Geography, Chinese Academy of Sciences, Urumqi, China

## Abstract

The hardened endocarp/stone is a defining feature of drupe fruits and is essential for seed protection, yet it poses significant challenges for fruit processing. Stone development is not an isolated process; rather, it is closely coordinated with seed and fruit growth through complex hormonal and genetic signaling networks. This review synthesizes advances in elucidating how these networks guide the developmental trajectory from early cellular specification in the ovary to lignification and programmed cell death (PCD). Following fertilization, a conserved transcriptional cascade is initiated in which embryo-derived signals activate MADS-box and other regulatory factors to determine endocarp fate. Subsequent upregulation of additional transcription factors/genes, such as NAC, MYB, PODs, LACs, and others, drives cell division and specialization, ultimately leading to activation of the phenylpropanoid pathway and the biosynthesis and deposition of lignin in endocarp cells, which is central to stone hardening. As fruit ripens, the lignified tissue undergoes PCD, forming a rigid protective structure. This developmental process is highly coordinated, and disruptions—whether genetic, hormonal, or environmental—can produce phenotypes such as split-pit, incomplete lignification, or altered stone thickness. Despite research progress, the spatial and temporal regulation of genes and their associated biochemical pathways during development remains poorly understood. The mechanisms by which metabolic and hormonal fluxes in the endocarp are directed from embryonic tissues to stony precursor cells, and how these processes contribute to differential lignification between endocarp and mesocarp tissues, require further investigation. This can be achieved through the integration of pangenomics, single-cell spatial transcriptomics, and precision gene editing.

## Introduction

Drupe fruits are characterized by a hardened, lignified endocarp/stonycarp, commonly known as a stone. Stone protects the seed but poses significant agronomic (seed germination) and consumer challenges (processing) [1]. Drupe fruits belong to different families and exhibit greater diversity in growth patterns. Most members belong to the Rosaceae (cherry, peach, plum, apricot, almond, and blackberries), Anacardiaceae (mango and pistachio), Oleaceae (olive), Rubiaceae (coffee), Arecaceae (coconut), and Rhamnaceae (Ber) family. Drupe fruits in the Rosaceae family typically follow a double-sigmoid growth curve, while pome fruits (apple and pear) exhibit a single sigmoid growth curve [2]. However, some drupe fruits, such as mango and ber, exhibit a single-sigmoid growth curve, suggesting that drupe growth patterns are not strictly conserved. Similarly, strawberry, a nondrupe accessory fruit, may show either single- or double-sigmoid growth depending on the variety [3, 4]. These findings collectively suggest that fruit growth patterns are not strictly linked to fruit type and may be influenced by additional physiological or genetic factors. Furthermore, the process of stone development is not universal or straightforward; it may vary even among drupe species. Recent comparative genomics and transcriptomics studies provide deep insights into drupe and nondrupe systems and offer new evolutionary perspectives on endocarp lignification.

Efforts to explore the molecular basis of stone development in fruits are increasing due to the growing demand for stoneless or small-stone fruits. Advances in sequencing, genome assembly, genetics, genomics, and transcriptomics have accelerated progress. Stone formation is a coordinated developmental program that begins with early tissue-fate specification and culminates with secondary-wall lignification and programmed cell death (PCD). Following fertilization, the fruit wall (pericarp) differentiates into three layers: the exocarp (epidermis), mesocarp (fleshy mesocarp), and endocarp (stone) [1]. Anatomical studies describe that the demarcation line between the tissues destined to become stony and those remaining fleshy is established very early, even before anthesis. In sour cherry, this demarcation was evident ~18 days before full bloom. The endocarp originates from a distinct group of precursor cells, consisting of one row of inner epidermal cells and three or four layers of pericarp cells. These cells are smaller, more densely packed, and exhibit a characteristic division pattern, forming a clearly demarcated ‘inner stony pericarp’ and ‘outer stony pericarp’ that are clearly distinct from the fleshy mesocarp [5]. These observations suggest that endocarp precursor cells are specified as a distinct lineage and that stone development is a well-coordinated process.

In drupes, the endocarp subsequently undergoes a coordinated developmental program involving cell fate specification, cell division and multiplication, secondary wall thickening, lignin deposition, and PCD, which together generate the rigid sclerenchymatous shell. In *Prunus* species like *Prunus persica* (Peach), lignin synthesis (lignification) takes 2–3 weeks [6–8]. In *Arabidopsis thaliana* and *P*. *persica*, *MADS-box* transcription factors such as *SHATTERPROOF* (*SHP*) and SEEDSTICK (*STK*) are upregulated in the endocarp postpollination and are critical for its specification [1, 6]. Subsequently, *NAC SECONDARY WALL THICKENING PROMOTING FACTOR1* (*NST1*) and *MYB* are activated, which in turn induce the suite of enzymes in the phenylpropanoid pathway responsible for lignin biosynthesis and secondary cell wall formation [6, 9]. The coordination of this network ensures the spatial and temporal precision of lignification, which is crucial for producing a fully developed stone.

In this context, the onset of lignification is tightly linked with the fruit growth phases, specifically stage II (lag phase) of the double-sigmoid growth curve. Notably, early-ripening cultivars have a shorter stage II than late cultivars, which is linked with the early start of endocarp lignification and enhanced sugar accumulation. These findings suggest that early cultivars may have high sugar contents and usually less developed seeds, likely due to a shorter stage II [10–12]. For example, early (Early Purple Guigne) and late (Downer) ripening cultivars of sweet cherry produced 15% and 100% viable seeds, respectively. Moreover, the late ripening cultivar stage II lasted twice as long (14 days as compared to 7) than the early cultivars [5]. These cultivar-specific changes within species indicate that the timing of endocarp lignification is developmentally flexible, and the interaction between endocarp lignification and fruit growth can be genetically modified. Consequently, for developing stoneless fruit, early cultivars may represent promising starting materials, as their naturally short lignification window can be further modified. Supporting this idea, disruption of lignification timing or tissue patterning resulted in phenotypes such as split-pit in peach, stoneless plum, and soft-endocarp apricot [1, 12, 13].

**Figure 1:**
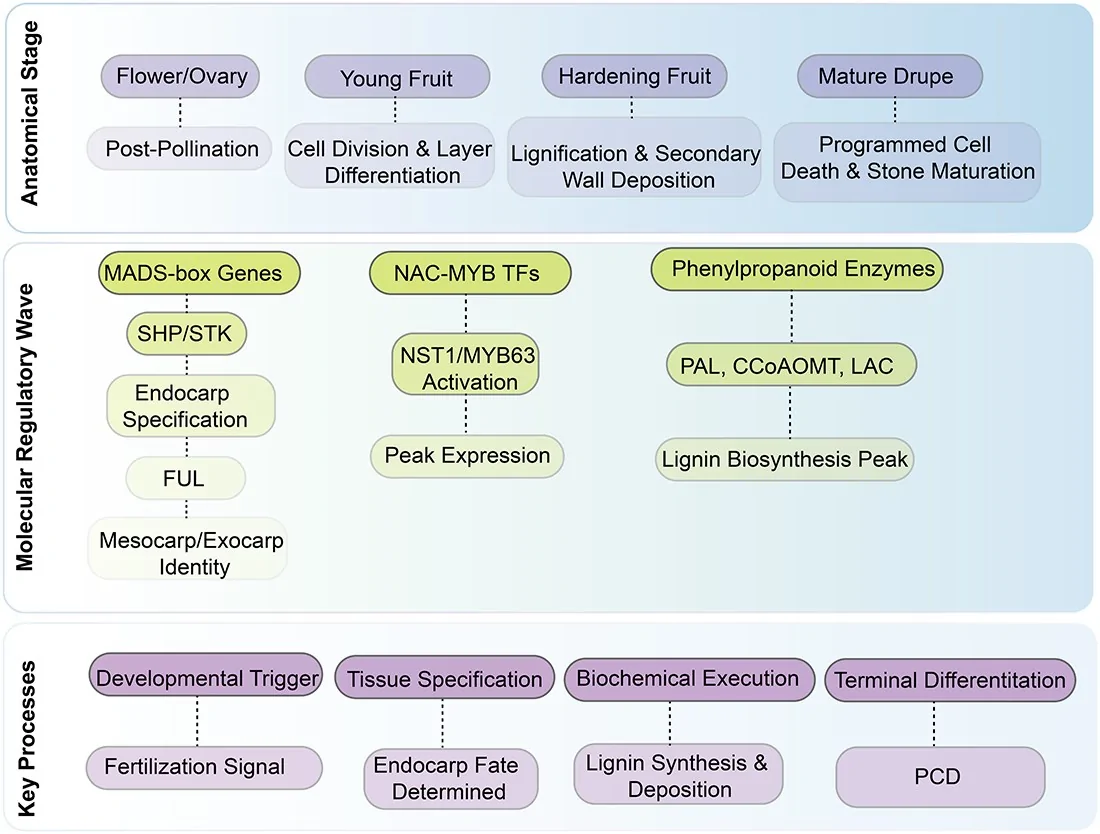
Overview of stage-wise genetic and molecular coordination of embryo-to-stone development in drupe fruits.

**Figure 2:**
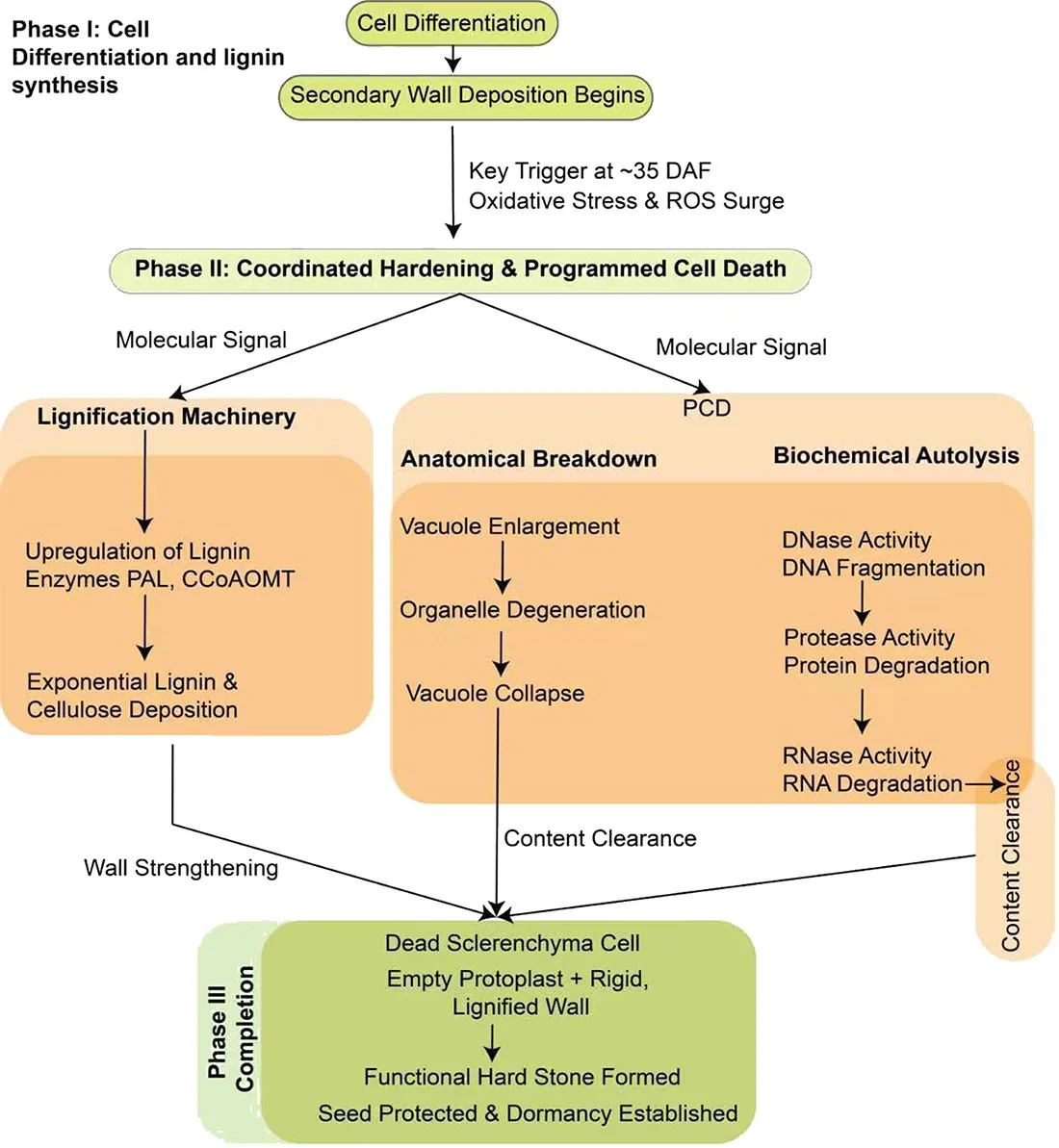
Detailed description of all phases of endocarp hardening at the anatomical and molecular level. The first phase begins with cell differentiation and secondary wall deposition. Lignification is driven by a coordinated regulatory network involving transcription factors that activate the phenylpropanoid pathway for monolignol biosynthesis, followed by oxidative polymerization mediated by peroxidases and laccases, leading to lignin accumulation and secondary wall thickening. In parallel, PCD is initiated, characterized by vacuolar expansion, organelle degradation, activation of hydrolytic enzymes, and onset of cellular autolysis. The coordination of lignification and PCD culminates in the formation of dead, lignified sclerenchyma cells, resulting in a rigid endocarp.

**Figure 3:**
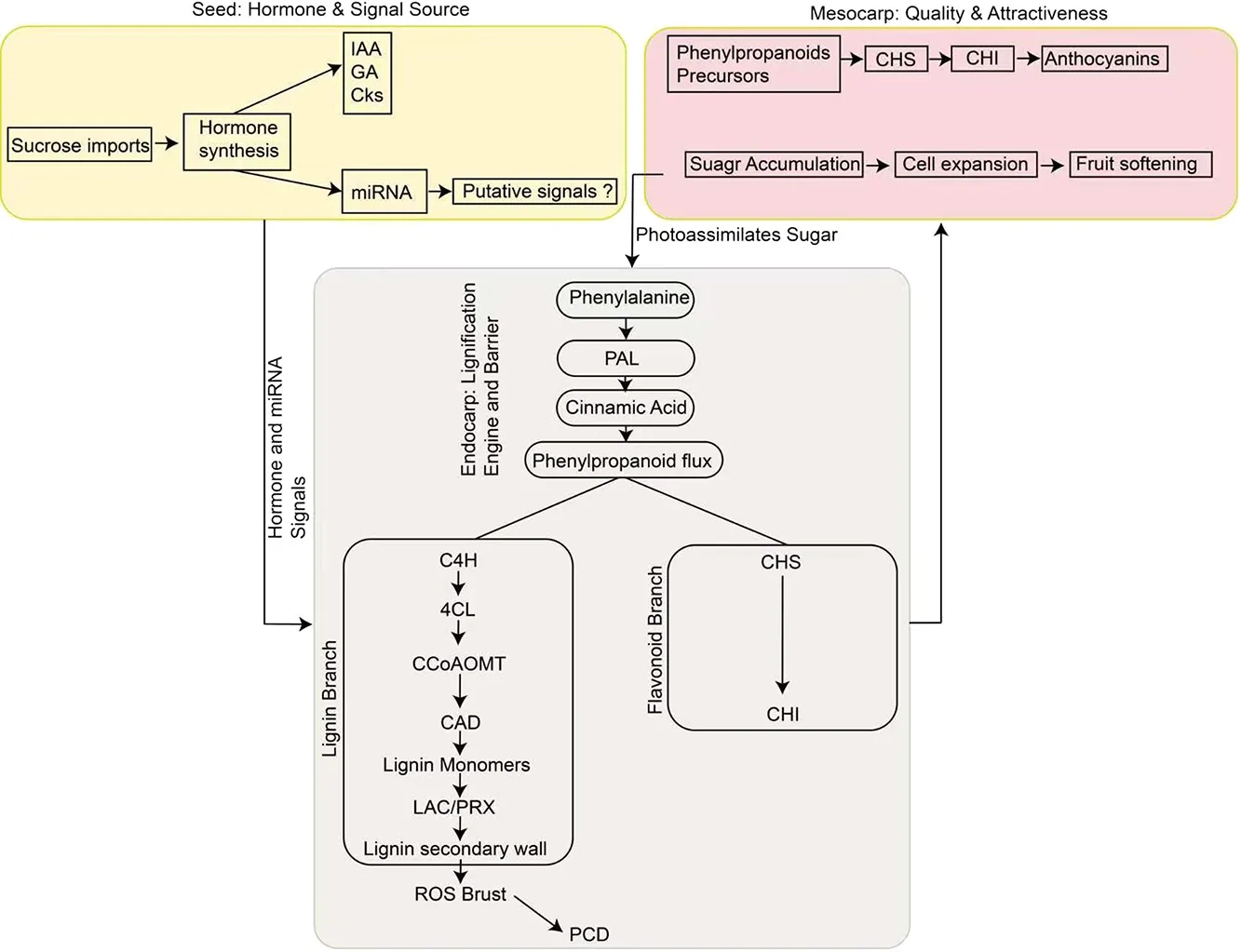
A conceptual model describing hormone signaling, phenylpropanoid metabolism, and endocarp hardening in drupe fruits. Seed and mesocarp above the yellow and pink boxes denote that these processes take place here.? Denotes still not confirmed.

Although many gene families have been proposed to influence stone development through lignin synthesis or endocarp hardening [14, 15], based on expression profiling. However, functional validation has been performed for only a few genes. Recently, comparative anatomical, lignin staining, and transcriptomic analyses have identified *ZjF6H1-3*, *ZjPOD*, and basic Helix-Loop-Helix (bHLH) transcription factors in Chinese jujube (*Ziziphus jujuba* Mill.) as key regulators of endocarp lignification [16], providing targets for breeding stoneless cultivars. A detailed overview of genes, hormones, and key enzymes involved in this process is provided in Tables 1–3, respectively. Despite this foundational knowledge, critical gaps persist in our understanding of stone development and its link with seed or fruit development. In particular, the initial signals that trigger the lignification network and the coordination of its timing with seed and mesocarp development remain poorly characterized [1, 7]. Therefore, this review article aims to: (i) summarize the current progress made in exploring the genetic and molecular basis of stone development in fruit crops; (ii) examine the interactions among seed development, stone, and fruit growth, and (iii) identify potential strategies/steps that need to be followed for developing stone-free drupe fruit without a seed viability or fruit quality penalty. To achieve these aims, three stages of stone development (lignin synthesis, accumulation, and PCD) are discussed, including their interdependencies and relative effects (Figs 1–3).

**Table 1:** Key genes and transcription factors regulating endocarp (stone) formation: pathways, roles, and functional evidence.

**Species**	**Genes/TFS**	**Role in stone formation**	**Pathway/process**	**Functional evidence**	**Reference**
Jujube (*Ziziphus jujuba*)	*ZjbZIP33*	Activates *ZjPRX1* expression by binding the G-box; promotes lignin formation in endocarp	Regulation of lignin biosynthesis	Yeast one-hybrid, dual-luciferase, EMSA; expression induced by IAA and GA3	[16]
	*ZjPRX1*	Catalyzes peroxidase-mediated lignin polymerization; major contributor to endocarp lignification	Lignin polymerization step	Overexpression increases lignin in jujube fruits and callus; OE Arabidopsis shows thicker secondary walls; high expression in endocarp	[16]
	*ZjF6H1-3, ZjPOD*, bHLH TFs	Promote lignin biosynthesis; contribute to endocarp hardening and stone formation	Lignin synthesis; phenylpropanoid pathway	Overexpression and silencing assays confirmed roles of *ZjF6H1-3* and *ZjPOD* in lignin accumulation. Promoter analysis showed bHLH-binding motifs, linking TFs to regulation of lignification genes	[23]
*Cornus officinalis*	PAL, C4H, CSE, CAD, CCR, POD, COMT, F5H	Regulate lignin biosynthesis driving endocarp sclerification	Phenylpropanoid and monolignol pathways	Early-stage genes (PAL, C4H, CSE, CAD, CCR, POD) are highly expressed in young fruit; late-stage genes (COMT, F5H) peak at ripening where lignin deposition is highest	[18]
Walnut (*Juglans regia*)	*JrMYB180*	Key regulator of GA3-induced lignification in the endocarp; acts as a hub gene in MYB regulatory network	Regulation of lignin biosynthesis during shell hardening	Strongly induced by GA3; validated by transient overexpression, subcellular localization, and qRT-PCR; identified as hub gene in coexpression network	[21]
	Multiple MYBs (total 207 identified)	Broad MYB family involvement in GA3-induced lignification	Transcriptional control of phenylpropanoid/lignin pathways	Transcriptome shows MYBs are the main regulators responding to GA3	[21]
	*JrLAC12-1*, *JrLAC12-2*, *JrLAC16*	Promote lignin accumulation during endocarp hardening	Lignin polymerization (late phenylpropanoid)	Strong induction after 44 DAF; expression correlates with lignin content; high expression in young tissues	[24]
	JrLAC family (37 members identified)	Multiple members contribute to endocarp lignification	Secondary cell wall formation	Nine JrLACs sharply upregulated after lignification onset; subfamily IV expansion	
Iron walnut (*Juglans sigillata*)	JsiFBP2, JsibHLH94	Endocarp formation in the early stage	Cell growth/early endocarp formation	Identified by tissue-specific expression and coexpression network	[27]
	JsiNAC56, JsiMYB78	Regulates lignification during late stage	Late stage of lignification		
Apricot (*Prunus armeniaca*)	NST1	Likely regulator of lignin deposition in endocarp; reduced expression explains soft, cleavable endocarp of ‘Liehe’ (LE)	Secondary wall thickening; regulation of phenylpropanoid/lignin pathway	Expression in LE only 13.7%, 2.8%, 9.4%, 82.5% of JG across stages; coexpression suggests NST1 regulates CAD	[20]
Peach (*Prunus persica*)	PpMYB63	Activates key laccase genes (PpLAC20 and PpLAC30) involved in lignin polymerization in endocarp	Regulation of lignin biosynthesis	Activates PpLAC20 and PpLAC30 promoters; homolog of AtMYB58/63	[15]
	PpLAC20, PpLAC30	Strongly expressed in endocarp; likely major contributors to lignin polymerization and endocarp hardening	Lignin polymerization	Expression profiling identifies them as key members; activated by PpMYB63; GUS staining shows tissue-specific expression pattern	[7]
	Homologs of Arabidopsis SHP, STK	Endocarp identity and lignification	Endocarp specification	Endocarp-specific expression	
	Homologs of Arabidopsis NST1	Promotes secondary wall thickening	Lignin biosynthesis	Endocarp-specific induction	
	Arabidopsis homologs of FUL	Negative regulator of lignification	Fruit tissue patterning	Higher in mesocarp/exocarp	
Huangguan plum (*Prunus salicina*)	PsPAL1, PsLAC7	Excessive lignification associated with hollowness and browning (HB)	lignin biosynthesis.	Both genes show strong upregulation in HB fruit during postveraison and ripe stages	[31]
Sweet cherry (*Prunus avium*)	PavSPL2, PavSPL9, miR156	Linked to the regulation of endocarp lignification during the transition from development to ripening	Lignin biosynthesis; anthocyanin pathway (ripening-related); miR156–SPL regulatory module	Coexpression networks connected PavSPLs with lignin biosynthetic genes • Auxin treatment reduced lignification and downregulated PavSPLs, supporting functional involvement	[10]

SHP, shatterproof; STK, seedstic; NST1, NAC secondary wall thickening promoting factor 1; CAD, Cinnamyl alcohol dehydrogenase

**Table 2:** Proposed roles, associated developmental stages, and downstream targets of phytohormones during stone formation.

Hormone	Role	Stage	Downstream targets	Crops	References
IAA	Induces expression of ZjbZIP33 and ZjPRX1; promotes lignin accumulation	Induction phase of endocarp lignification	ZjbZIP33 → ZjPRX1	Jujube	[16]
GA3	Induces expression of ZjbZIP33 and ZjPRX1; enhances lignin formation	Induction phase of lignification	ZjbZIP33 → ZjPRX1		
GA3	Accelerates early-stage endocarp lignification; induces MYB regulatory module	Early stages of shell hardening	JrMYB180 and other MYBs	Walnut (*Juglans regia*)	[21]

**Table 3:** Functions, pathway steps, and genetic regulation of key enzymes during stone formation.

**Enzyme**	**Function**	**Pathway step**	**Gene**	**Stage/Localization**	**Crops**	**References**
Class III peroxidase	Catalyzes oxidative polymerization of monolignols into lignin	Final lignin polymerization step	ZjPRX1	Highly expressed in endocarp; cytoplasmic localization	Jujube	[16]
F6H	Participates in monolignol biosynthesis	Lignin synthesis branch of phenylpropanoid pathway	ZjF6H1-3	Higher expression in endocarp of stone-forming cultivar		[23]
POD	Catalyzes oxidative polymerization of monolignols into lignin	Final lignin polymerization step	ZjPOD	Strongly expressed in lignifying endocarp of stone cultivar		
CAD	Converts cinnamyl aldehydes to monolignols; key for lignin monomer production	Monolignol biosynthesis in phenylpropanoid pathway	CAD	Expression and activity dramatically lower in LE; matches reduced endocarp lignification	Apricot	[20]
Laccase	Catalyzes monolignol polymerization, essential for lignin formation	Lignin polymerization	*PpLAC20*, *PpLAC30*	Highly expressed in endocarp; GUS staining shows expression in stems, siliques, seed coat	Peach	[15]
PAL	Initiates phenylpropanoid pathway	Phenylpropanoid entry step	PAL	Induced during lignification		[7]
C4H	Converts cinnamate derivatives	Early lignin pathway	C4H	Endocarp-specific induction		
CCoAOMT	Monolignol modification	Mid-lignin pathway	CCoAOMT	Strong induction		
Peroxidase	Lignin polymerization	Polymerization	POD	Endocarp localized		
Laccase	Monolignol polymerization	Polymerization	LAC	Endocarp lignification stage		
CSE	Converts caffeoyl shikimate to caffeic acid and shikimate; essential for lignin biosynthesis	Early monolignol biosynthesis	Arabidopsis Homologs of CSE	Strong induction in endocarp at 50–55 DAFB; expression markedly higher than mesocarp; protein detected in endocarp		[26]
Laccase	Polymerizes monolignols to form lignin	Lignin polymerization	JrLAC12-1, JrLAC12-2, JrLAC16	Strongly induced after 44 DAF; high expression in young stems; correlates with lignin increase	Walnut	[24]
CCoAOMT	O-methylation of caffeic acid derivatives contributing to lignin monomer synthesis	Lignin biosynthesis (methylation step)	DiCCoAOMT1	Endocarp-specific expression; increases progressively during fruit development	*Davidia involucrata*	[51]
PAL	Converts phenylalanine into cinnamic acid; initiates phenylpropanoid metabolism	Step of lignin biosynthesis	PsPAL1	Elevated enzyme activity and gene expression in HB fruit during postveraison stages	*Prunus salicina*	[31]
Laccase	Catalyzes oxidative polymerization of monolignols to form lignin polymer	Final polymerization step of lignin biosynthesis.	PsLAC7	Strongly upregulated in HB fruit at veraison and ripe stages		
F6H	Participates in monolignol biosynthesis	Lignin synthesis branch of phenylpropanoid pathway	ZjF6H1-3	Higher expression in endocarp of stone-forming cultivar		
Lignin biosynthesis enzymes (PAL, C4H, CSE, CAD, CCR, POD, COMT, F5H)	Lignin biosynthesis enzymes (PAL, C4H, CSE, CAD, CCR, POD, COMT, F5H)	Phenylpropanoid and terminal lignin synthesis		Early enzymes peak in young fruit; COMT and F5H peak during ripening when lignin deposition is maximal	*Cornus officinalis*	[18]

Abbreviations: POD, peroxidase; F6H, flavonoid 6-hydroxylase; CSE, caffeoyl shikimate esterase; Caffeoyl- CCoAOMT, CoA O-methyltransferase; CAD, cinnamyl alcohol dehydrogenase; PAL, phenylalanine ammonia-lyase; C4H, cinnamate 4-hydroxylase; CCR, cinnamoyl-CoA reductase; COMT, caffeate O-methyltransferase.

## Lignin biosynthesis

Initial studies on lignin synthesis were conducted in model plants such as *A. thaliana*. This program is initiated after fertilization and proceeds in a precise temporal sequence, beginning with cell-fate determination and ending with the complex biochemical process of lignification. A suite of MADS-box transcription factors controls the first phase of endocarp development. In peach, homologs of *SHP* and *STK* are upregulated explicitly in the endocarp postpollination, defining the tissue destined to become the stone [6]. Subsequently, *FRUITFUL (FUL)* is predominantly expressed in the mesocarp and exocarp, differentiating the lignification boundary and confining stone development [1]. The specification of the endocarp and its subsequent lignification are controlled by a core genetic program, highly conserved from the model plant *Arabidopsis* to commercially important *Prunus* species [1]. Following this differentiation, the endocarp undergoes lignification, forming the stone. *Prunus* species follow a double-sigmoid fruit growth pattern, and a slowdown in growth is tightly linked with endocarp lignification and hardening [1, 17]. This change in growth likely reflects increased metabolic demand for secondary wall formation and lignin biosynthesis [13]. These genes play primary roles in lignification activation. Similar stage-specific lignification has been reported in *Cornus officinalis* (Japanese cornel or Japanese cornelian cherry), where early-stage genes such as *PHENYLALANINE AMMONIA-LYASE* (*PAL*), *CINNAMATE-4-HYDROXYLASE* (*C4H*), *CAFFEOYL-SHIKIMATE ESTERASE* (*CSE*), *CINNAMYL ALCOHOL DEHYDROGENASE* (*CAD*), *CINNAMOYL-COA REDUCTASE* (*CCR*), and *PEROXIDASE* (POD) peak their expression at young fruit development stages, while *CAFFEIC ACID O-METHYLTRANSFERASE* (*COMT*) and *FERULATE 5-HYDROXYLASE* (*F5H*) attain it at the late ripening stage when lignin accumulation was highest [18].

Subsequently, the transcriptional cascade initiates secondary cell wall formation, involving genes such as *NST1*, *NACs,* and *MYBs* [7]. This cascade directly targets *PAL* activation, encoding a key enzyme in the phenylpropanoid pathway indispensable for lignin biosynthesis [7, 19, 20]. A similar regulatory cascade also occurs in apricot, where reduced expression of *NST1* results in the soft, cleavable endocarp of the ‘Liehe’ variety, likely due to its role in regulating downstream genes like *CAD* [20]. In walnut (*Juglans regia*), gibberellic acid (GA3)-responsive regulators such as *JrMYB180* and several other *MYBs* influence early shell hardening, coupling hormonal signaling with transcriptional control of lignin biosynthesis [21]. More relevant studies in other crops are mentioned in Table 1. This pathway requires key enzymes, including PAL, C4H, 4CL, Caffeoyl-CoA O-methyltransferase (CCoAOMT), and CAD, which collectively drive lignin synthesis and deposition [22]. Studies on jujube provide deep insights, confirming that genes such as *ZjF6H1-3* (ferulic acid 5-hydroxylase) and class III peroxidases (*ZjPOD*) are major contributors to lignin biosynthesis and endocarp hardening [23]. Peach endocarp lignification also depends on laccases (*PpLAC20* and *PpLAC30*), which are directly activated by *PpMYB63* [15], while in walnut, key laccases such as *JrLAC12-1*, *JrLAC12-2*, and *JrLAC16* are expressed during late lignification [24]. Detailed information on other key enzymes in different species that play critical roles in lignin synthesis is listed in Table 3.

GA_3_ has also been shown to be a key inducer, accelerating early-stage lignification in walnut by inducing a regulatory module of MYB transcription factors, including the hub gene *JrMYB180* [21]. Similarly, in jujube, both indole-3-acetic acid (IAA) and GA_3_ induce the expression of *ZjbZIP33*, which leads to upregulation of *ZjPRX1* [16]. This genetic and hormonal interplay contributes to the precise spatiotemporal control of lignification. Mostly, the process starts 4–7 days after bloom along the fruit suture and funiculus, progressively spreading throughout the endocarp within 2–3 weeks [1, 6, 7]. The specific roles of major hormones, such as IAA and GA3, in modulating this process in other species are detailed in Table 2.

At the molecular level, *P*. *persica* exhibits transcriptional dynamics highly similar to those of *Arabidopsis*. The *MADS-box* genes *SHP* and *STK* show tissue-specific expression in endocarp and seed and are upregulated postpollination, where they specify lignifying tissues [7, 14]. Their expression declines at the onset of lignin biosynthesis, coinciding with the upregulation of the NAC-domain transcription factor *NST1* and other genes involved in secondary wall formation and phenylpropanoid metabolism [25]. In contrast, *FUL* showed high expression in the mesocarp and exocarp, specifying the lignification boundary and preventing ectopic hardening [7]. Lignin and flavonoid biosynthesis pathways share precursors with the phenylpropanoid route, leading to metabolic competition. During early fruit development, genes of both paths are transcriptionally active across pericarp tissues; however, in the endocarp, lignin biosynthetic genes are selectively upregulated while flavonoid pathway genes are repressed, ensuring carbon flux directed toward lignification [1, 7, 26]. Flavonoid synthesis is dominant in mesocarp and exocarp and plays a significant role in pigment formation, flavor development, and defense mechanisms. This spatial metabolic differentiation endorses the dual role of the phenylpropanoid pathway [26]. Earlier studies suggested that the process of endocarp determination and differentiation is conserved [1]. Recent studies on sweet cherry, jujube, and iron walnut have identified additional regulatory layers involving SQUAMOSA promoter-binding protein-like (*SPLs*), *miR156*, *FBP2*, *bHLH94*, and lineage-specific *MYBs* [11, 23, 27], indicating that specific regulatory mechanisms may vary among species.

## Lignin accumulation preceding stone hardening

The onset of lignification is associated with increased tissue hardening, suggesting a coordinated process between lignin accumulation and endocarp hardening [7]. After monolignol biosynthesis, these precursors are transported to the cell wall and oxidatively polymerized by laccases and peroxidases, leading to rapid secondary wall thickening and loss of cell wall plasticity, thereby marking the transition from a soft to a hardened endocarp.

The process is spatially coordinated; while phenylpropanoid genes are initially induced in all pericarp layers, the endocarp sees a specific induction of the lignin branch and repression of the competing flavonoid pathway, ensuring resources are channeled toward lignification [1]. This suggests that it is a spatiotemporal coordinated process. This is supported by the coordinated expression of lignin biosynthesis enzymes (PAL, C4H, CSE, CAD, CCR, POD, COMT, F5H) in *C*. *officinalis*, where early-stage genes are highly expressed in young fruit and late-stage genes peak during ripening when lignin deposition is maximal [18]. Moreover, some hormones play a regulatory role in endocarp hardening. In walnut, the content of IAA and the expression of auxin-responsive genes like *AUX/IAA* members (*JrIAA9, JrIAA16, JrIAA27*) were closely linked with the timing of endocarp hardening, suggesting their role in modulating this process [28].

Natural and cultivated variants support the central role of the coordinated regulatory network. In the ‘Stoneless’ plum (*P*. *domestica* var. *Sans Noyau*), proper endocarp specification was lacking and resulted in the production of a partially exposed seed. Interestingly, secondary metabolism genes were upregulated in this mutant, implying that lignification capacity is maintained even in the absence of a defined endocarp layer. The failure is the absence of endocarp tissue specification, not a disruption of the lignification process, suggesting that the initial genetic regulation for layer formation is distinct from the subsequent biochemical hardening [13]. Thus, stone development can be considered as a two-step genetic cascade comprising: (i) early specification and proliferation of endocarp precursor cells into distinct inner and outer stony layers after fertilization, and (ii) subsequent activation of the lignin biosynthetic and polymerization machinery that converts these prepatterned tissues into a rigid, lignified shell. Thus, the distraction in the first step prevents stone formation even when lignin biosynthetic capacity is present (as in stoneless plum), and an alteration in the second step leads to a properly specified but partially hardened endocarp. For example, the ‘split-pit’ phenotype in peach, accompanied by low *SHP* and high *FUL* expression, suggests that dysregulation of the boundary between the endocarp and fleshy fruit tissues can lead to defective stone formation [6].

More phenotypic diversity in the fruit of different species well explains the fine regulation of stone development. Almonds have greater diversity in shell thickness, hardness, and brittleness [29]. In peach, the ‘split-pit’ phenotype results from incomplete endocarp fusion along the suture, mostly seen in cultivars with rapid fruit expansion before full lignification [30]. Excessive lignification in ‘Huangguan plum’ resulted from the upregulation of *PsPAL1* and *PsLAC7,* which led to physiological disorders like browning and fruit hollowness [31]. These studies suggest that increased lignin accumulation can negatively influence fruit quality and market demand (for details Tables 1 and 3).

In summary, the conserved *SHP*–*STK*–*FUL*–*NST1* transcriptional module is the central regulator of endocarp fate and lignification. The balance between lignin and flavonoid biosynthesis is essential for proper seed, stone, and fruit growth and development. However, the signal that triggers this regulatory cascade and coordinates it with seed and mesocarp development is not exactly known, offering a major frontier for future drupe biology and breeding research.

## Programmed cell death

PCD is the final step in the stone-hardening process in drupes. PCD does not initiate lignin deposition but acts as a terminal developmental program that converts lignified endocarp cells into rigid, metabolically inactive sclereids. Anatomical studies in different crops, such as *C. officinalis*, peach, walnut, and *Argania spinosa* (Argan), suggest a conserved pattern of PCD [18, 32]. First, endocarp parenchyma cells differentiate, then expand and initiate secondary wall deposition, and finally progressively lose organelles, collapse their vacuoles, and die. In *A. spinosa*, the sclerenchyma cells acquire thick lignified walls with a porous structure before the protoplast is eliminated, indicating a built-in cell death program that produces the rigid barrier responsible for exogenous dormancy [32]. Transmission electron microscopy in peach reveals that vacuole enlargement begins as early as 8 days after anthesis, organelles degenerate by ~40 days, and the cell wall thickens as PCD proceeds [33]. Similar correlations between lignin accumulation, stone cell formation, and PCD were documented in walnut endocarp development [27].

At the molecular level, three processes converge to trigger and complete PCD: lignification, oxidative stress, and activation of hydrolytic enzymes. Lignin biosynthesis accelerates during early endocarp development, supported by high activities of PAL, CCR, CAD, POD, and other enzymes. The importance of specific enzymes in this final stage is highlighted by studies showing that laccases, which catalyze the final polymerization of lignin, are strongly expressed in the endocarp hardening of crops like peach (*PpLAC20*, PpLAC30) and walnut (*JrLAC12-1*, *JrLAC12-2*, *JrLAC16*) [15, 24]. Moreover, the roles of different enzymes in different plants are further elaborated in Table 3. As lignin deposition increases, reactive oxygen species (ROS) production rises, and antioxidant enzyme activity declines. In peach, downregulation of MnSOD, MDAR, LGL, and β-CAS after 35 days after flowering (DAF) caused accumulation of H₂O₂ and superoxide, which are known signals for lignin-biosynthesis genes and PCD initiation [8]. Following secondary wall thickening and lignin polymerization, PCD removes the cellular contents, terminates wall remodeling, and compacts the tissue, which together lock the mechanical properties of the endocarp. This oxidative burst appears to serve as a developmental trigger, shifting the endocarp into a cell-death mode. Concurrently, inhibition of the flavonoid pathway and redirection of metabolic flux toward monolignol production further accelerate secondary wall formation.

Once PCD is initiated, a suite of hydrolytic enzymes completes the autolytic phase. In peach, DNase activity rises first (peaking ~52 DAF), followed by RNase, acid proteases, and neutral proteases, which sharply degrade soluble proteins after 35 DAF as the vacuoles collapse and phenolic substrates accumulate [33]. These enzymatic waves dismantle the cytoplasm while lignification completes the secondary wall, leaving behind an empty, thick-walled sclereid. In coordination with cellulose synthesis directed by genes such as *PpCesA1* during late hardening [34]. Through elimination of the protoplast, cessation of cell wall metabolism, and collapse of intercellular spaces, PCD transforms the lignified endocarp into a dense, impermeable, and mechanically stable barrier. The systematic progression of oxidative stress, lignification, and PCD culminates in a fully hardened endocarp that safeguards the seed and defines the anatomy of drupe fruits. In walnut and jujube, integration of PCD with transcriptional and enzymatic control was similar, proposing a conserved terminal step in stone maturation [16, 27].

## Seed, stone, and fruit development interactions

Coordinated signals exchanged among the seed, the endocarp, and the fleshy fruit ensure complete development of the stone, and their developmental processes work together rather than separately. Fruit development is started after fertilization and is controlled by signals derived from the developing embryo and endosperm, indicating the key regulatory role of the seed in orchestrating fruit and stone development [35]. Transcriptomic studies in sweet cherry, peach, and other drupes exhibit that each tissue follows a distinct but interconnected trajectory [11, 36]. Seed is the central regulator of this hierarchy. Its rapid postfertilization growth and the early expansion of the endosperm accord with nucellus reabsorption and lead to the onset of endocarp lignification [1, 5]. Fertilized seeds initiate the genetic program essential for stone identity [1, 6]. Seed-derived signals play a central role in initiating the stone development program. Auxins, cytokinin, and gibberellins contribute to early seed–pericarp communication, while auxin acts directly, and the other hormones work indirectly [36]. Auxin synchronizes the development of the endosperm and seed coat, acting as a unifying signal that coordinates all seed components [37]. Auxin produced in the endosperm is anticipated to move toward the embryo and seed coat, facilitating intertissue communication [38]. The exact mechanism of hormone transportation is not exactly known. Disorders in early seed development lead to defects such as fruitlet abscission [39] and the stoneless plum [13]. This demonstrates how failure of seed-dependent signaling results in incomplete endocarp specification and a partially exposed seed.

Endocarp lignification controls the pace and pattern of fruit growth. In peach and sweet cherry, the period of stone hardening is accompanied by a decrease in mesocarp expansion [1, 17]. The potential explanation for the decrease in mesocarp expansion is a shift of sugars and metabolic resources toward lignin biosynthesis in the endocarp [13]. If there is no proportional coordination between lignification and mesocarp expansion, the developing stone cannot bear the internal pressure, leading to disorders such as split-pit [6, 30]. This demonstrates that the stone not only protects the seed but also controls the fruit’s growth. Recent findings suggest that the lignified endocarp may act as a dynamic barrier or a source of signals that influence ripening. Exogenous application of auxin on the midseason cultivar (Lapins) of sweet cherry induced lignification and pigmentation after 3 weeks of treatment at 46 days after full bloom. While application of p-IBA (inhibitor) reduced lignin accumulation and transiently increased anthocyanin accumulation, suggesting a functional link between stone hardening and the onset of ripening processes [10]. Auxin, GA, abscisic acid (ABA), and cytokinins (CKs) found in the seed and the pericarp, regulate these processes. In peach, strong CK signaling was noticed during and after lignification in the seed tissues, contrasting with mesocarp expression profiles [36]. In Peach, exogenous GA application at 50 ppm induced parthenocarpy (73.4%) and high fruit set, while the higher concentration (500 ppm) of GA promoted harder stones and sclerification [40]. This highlights the direct influence of seed-derived GA on stone development. Recently, this has been elucidated in grape, where GA limits lignin synthesis in the seed-stone through a multilevel cascade. VvSLR1 (DELLA protein), a key repressor in GA signaling, works together with *VvWRKY26* to inhibit expression of *VvmiR397a*, thereby reducing suppression of its target *VvLAC4* (a laccase involved in lignin polymerization), leading to lignin accumulation. On the other hand, exogenous GA promoted degradation of VvSLR1, releasing *VvWRKY26* to activate *VvmiR397a* transcription, which in turn suppressed *VvLAC4* expression and decreased lignin accumulation in the seed-stone [41]. This regulatory network provides a theoretical basis framework for understanding how seed-derived GA signals can influence endocarp lignification in drupes.

‘Celeste’ (early cultivar of sweet cherry) displayed higher auxin and ABA activity at the transition from development to ripening. While ‘Regina’ (late cultivar) retained higher levels of amino acids and chlorophyll, it showed delayed ripening [11]. These discrepancies in maturity time reflect distinct roles of hormones. These results align with earlier findings, as auxin application promotes early ripening [42] and GA delays ripening [43]. The presence of large quantities of IAA, GA, and ABA in seed tissues [44] highlights their role as hormonal regulators of fruit ripening. In sweet cherry, miRNA–hormone–transcript network analysis revealed that SPL transcription factors, the primary targets of miR156, contribute to developmental differentiation between early- and late-cultivar stages and are directly linked to differences in seed and mesocarp development. These SPL genes connect hormonal signals with downstream lignin and inositol-related pathways, suggesting regulatory pathways previously unknown in nonclimacteric ripening [11]. This study proposes that small RNAs are also involved in this coordination. Moreover, the phenylpropanoid pathway supplies both lignin and flavonoids, but both are allocated differently across tissues. Endocarp cells suppress the flavonoid pathway to channel precursors toward lignin [7], while the mesocarp supports flavonoid biosynthesis for pigmentation, flavor, and defense [26]. At the transition from development to ripening, this specialization deepens as sugars accumulate in both the seeds and the mesocarp. Then, hormone-regulated transcription triggers fruit ripening onset [45]. These studies further support the concept of sophisticated division of labor.

In summary, seed maturity, endocarp lignification, and mesocarp development operate in an interdependent, coordinated manner rather than as separate developmental modules. Most findings support their functional integration, yet most are based on assumptions drawn from individual tissue analyses rather than on direct mechanistic studies of tissue-to-tissue signaling. Building a logical network of this cascade of interactions will require further experimental work to track molecular signals across all three developmental domains.

## Research gaps and future perspectives

Stone development can be regarded as two distinct steps of a genetic cascade: first, the specification of stone precursor cells before fertilization, and second, the initiation of lignification. Exploring these two phases can provide two viable strategies for developing stoneless cultivars: (i) disrupting the developmental process of precursor cells and (ii) blocking lignification. Therefore, future research should focus on identifying cell-specific regulators for each step to enable precise editing without compromising other agronomic traits. Consequently, deeper insights into stone development and its interactions with mesocarp tissues and seed-derived signals are urgently needed. Synthesis of published comparative genomic, transcriptomic, and anatomical studies suggests that, beyond individual species studies, comparative genomics offers a logical way to differentiate highly conserved mechanisms of endocarp lignification from lineage-specific regulatory innovations. Orthogroup-based comparisons across drupe-forming Rosaceae, sclereid-forming relatives, nonstone Rosaceae, and other stone-forming species outside of Rosaceae suggest that stone development primarily depends on the selective retention and coordinated regulation of secondary cell wall and lignification gene families, rather than the evolution of novel biochemical pathways.

The primary unresolved question is to detect the initial signal that triggers endocarp specification. The exact nature of the first trigger, whether it is a hormone gradient, peptide, or small RNA-like, remains unclear. Although embryo- or endosperm-derived auxin gradients are hypothesized as the initiating trigger for endocarp fate specification, but experimental confirmation is absent. Answering this fundamental question necessitates high-resolution, spatiotemporal ‘omics’ of the fertilized ovary, ideally combined with single-cell transcriptomics, proteomics, and spatial metabolomics to identify the earliest precursors/communication signals before anatomical differentiation becomes visible. These approaches have been successfully applied in *Arabidopsis* root [46] and the shoot apical meristem [47]. The second major question involves understanding how three phases (Lignin biosynthesis, accumulation, and PCD) of stone development are temporally and spatially coordinated with seed development and the onset of fruit ripening. A recent single-cell spatiotemporal RNA sequencing study in the pitaya pericarp [48] explained how the senescence process is initiated and which tissues and cell types underwent more changes. Performing a similar type of single-cell spatiotemporal study in drupe fruit would allow reconstruction of differentiation trajectories for seed, endocarp, and mesocarp tissues and test the hypothesis that the lignified endocarp modulates hormone and signal flux between tissues, as previously suggested [10]. Furthermore, integration of scRNA-seq with spatial transcriptomics and single-cell metabolomics will facilitate *in situ* tracing of lignin precursors, metabolomic flux, and hormonal crosstalk, thereby providing direct evidence for intertissue coordination rather than correlative observations.

Beyond development signaling, the genetic basis of natural variation in stone traits is less studied. The complex cascade of events in stone development includes multigene families of transcription factors (NACs, MYBs) and biosynthetic enzymes, yet the majority of NACs and MYBs are not functionally characterized. Genetic variations (single-nucleotide polymorphisms (SNPs); single base pair change) and structural variations (SVs; ≥50 bp change) might be key drivers of phenotypic diversity in shell thickness, hardness, and disorders such as split-pit. As compared to SNPs, SVs explain a greater proportion of phenotypic variation, as at least one-third of known domestication alleles are SV-driven, making them essential for understanding the genetic basis of adaptation and trait evolution [49]. Therefore, using a pangenome as a reference rather than single genomes will be essential to capture this hidden diversity and reduce reference bias [50]. We surmise that the construction of a comprehensive drupe fruit pangenome coupled with SV-aware Genome-wide association studies (GWAS) can identify high-impact alleles responsible for both beneficial traits and deleterious mutations.

**Figure 4:**
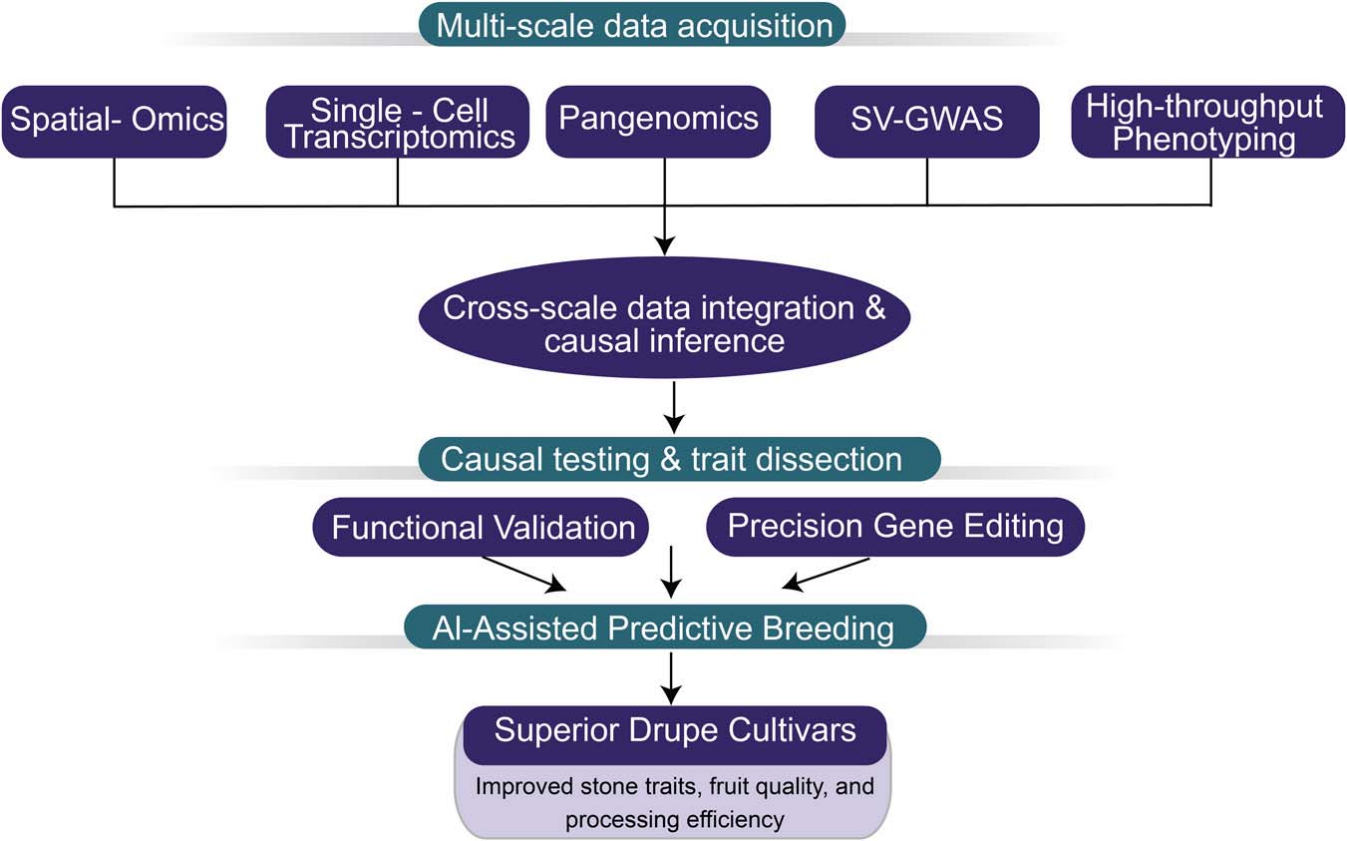
An integrated multi-omics and AI-driven framework for developing superior drupe cultivars. This detailed strategy will enable the generation of superior drupe cultivars.

A further bottleneck for translational breeding is the limited functional validation of key genes. Precision gene editing of master regulators such as *SHP* or *NST1* will clarify their pleiotropic effects on seed viability, fruit development, and quality. This approach would provide mechanistic insights into the phenotypes observed in natural variants like the stoneless plum mutant, and the thin, soft endocarp of the apricot cultivar ‘Liehe’ [12, 13]. Ultimately, future progress will depend on integrating pangenomics, scRNA-seq, spatial omics, gene editing, and AI-assisted predictive breeding into a unified network to fully elucidate the mechanisms of stone development (Fig. 4). This integrated approach will not only explore the full mechanism of stone development but also accelerate the rational design of the genome to develop superior drupe fruit.
